# The Use of MAGE C1 and Flow Cytometry to Determine the Malignant Cell Type in Multiple Myeloma

**DOI:** 10.1371/journal.pone.0120734

**Published:** 2015-03-20

**Authors:** Kirsty Wienand, Karen Shires

**Affiliations:** 1 Division of Haematology, University of Cape Town, Cape Town, South Africa; 2 Division of Haematology, National Health Laboratory Services/Groote Schuur Hospital, Cape Town, South Africa; French Blood Institute, FRANCE

## Abstract

The malignant cell phenotype of Multiple Myeloma (MM) remains unclear with studies proposing it to be either clonotypic B or proliferating plasma cells. Cancer/testis antigen MAGE C1 is being extensively studied in MM and it has been suggested that it is involved in the pathogenesis of the cancer. Therefore, we report on the use of MAGE C1 to determine the malignant cell phenotype in MM using flow cytometry. Bone marrow aspirate (BM) and peripheral blood (PB) was collected from twelve MM patients at diagnosis, as well as three MM disease-free controls. Mononuclear cells were isolated using density-gradient centrifugation, and stabilized in 80% ethanol, before analysis via flow cytometry using relevant antibodies against B cell development cell-surface markers and nuclear MAGE C1. MAGE C1 expression was observed consistently in the early stem cells (CD34^+^) and early pro-B to pre-B cells (CD34^+/−^/CD19^+^), as well as the proliferating plasma cells in both the MM PB and BM, while no expression was observed in the corresponding control samples. Monoclonality indicated a common origin of these cell types suggesting that the CD34^+^/MAGE C1^+^ are the primary malignant cell phenotype that sustains the downstream B cell maturation processes. Furthermore, this malignant cell phenotype was not restricted to the BM but also found in the circulating PB cells.

## Introduction

Multiple Myeloma (MM) is a haematological malignancy, characterised by the presence of monoclonal immunoglobulin (Ig) in the peripheral blood (PB) and large numbers of neoplastic plasma cells in the bone marrow (BM) [1–3]. Although, the disease mechanism responsible for the malignant phenotype of MM remains unclear, studies have suggested that it may be a two-compartment model comprising of both actively dividing and non-dividing cells which contribute to the disease characteristics [4–7]. The precursor cell type responsible for disease initiation remains the most contentious issue, with some studies supporting the theory that it is a pre-B cell (CD138^-^) capable of self-renewal that feeds the growing population of non-dividing plasma cells, while others favour the idea that the disease initiating cell is solely a plasma cell (138^+^) that is capable of regaining self-renewal characteristics [5,8–10].

While still controversial, the largest numbers of studies seem to favour the theory that clonotypic B (CD138^-^) cells are the precursor cells in MM [5,10–11]. However, the phenotypic profile of malignant clonotypic B cells, linked to disease initiation, varies between studies indicating that these cells resemble CD19^+^/CD27^+^/CD38^-^ memory B cells or a slightly less differentiated memory B-lymphocyte (CD20^+^/CD27^+^/CD34^−^/CD138^−^) as well as B cells with haematopoietic stem cell-surface characteristics (CD34^+^/CD19^+/−^) [5,8,10,12]. Furthermore, what stage in development clonotypic B cells become malignant is unclear, with studies suggesting that clonotypic B cells originate in the BM (CD34^+^/CD19^+/−^) or from the lymphatic organs (memory B cell) migrating to the BM giving rise to malignant plasma cells [5,8,10]. Identification and characterization of the malignant cell type in MM is important not only in understanding the role of the clonotypic B cell in the pathogenesis and disease specific biology of the cancer, but for effective treatment management of MM.

In the search for more answers, a group of genes that are actively being studied in MM are cancer/testis antigens (CTAs) [6,13–15]. These genes show highly restricted expression, with only testis tissue showing expression in all normal tissues thus far tested (including PB and BM) and yet a very strong link to malignant cell types in a multitude of cancers [15–16]. MAGE C1 is the most commonly expressed CTA in MM, with 85% to 100% of symptomatic MM patients expressing this antigen alone or with at least one other CTA [15,17]. Additionally, expression of MAGE C1 is not limited to the stage of the cancer of MM [6,15,17]. Several groups have suggested a direct role of this antigen in MM disease pathogenesis with Andrade *et al*. [17] and Atanackovic *et al*. [18] suggesting that MAGE C1 expression is a primary event in pathogenesis and may play a role in initiating abhorrent plasma cell proliferation in some MM cases [6,14,19–20]. Although studies are limited at this stage, it is thought that MAGE C1 plays a role in cell-cycle progression and is important for MM cell survival [19–20]. As MAGE C1 seems to play a role in the early development of MM, we used MAGE C1 antibodies in a flow cytometric approach to link the abhorrent expression of this CTA to a specific stage in the B cell maturation process in order to identify the primary malignant cell phenotype in MM.

## Materials and Methods

### Patient population and cell preparation

The study population consisted of twelve newly diagnosed, untreated symptomatic MM patients (as defined by the WHO classification) who were referred for BM biopsy at the Haematology Division at Groote Schuur hospital, Western Cape, South Africa (Table 1). Three healthy adult individuals (negative for any haematological diseases via immunocytochemistry and microscopy) who were referred for BM biopsy, as donors for allogeneic transplants, were also included in the study as controls (Table 1). HIV positive as well as dual-cancer pathology patients were excluded from the study. No specific MM subtype was selected for analysis. Ethics approval (HREC REF: 194/2012) was granted for this study from the Human Research Ethics Committee University of Cape Town and all participants provided written informed consent.

**Table 1:** Summary of MM patient characteristics at time of diagnosis.

MM patient/Donor number	Sex, Age^1^	Stage of Disease at diagnosis (DS)^2^	CRAB^3^	Igκ/Igλ^4^
MM1	F, 56	IIa	AB	IgGκ
MM2	F, 66	IIa	RAB	IgGλ
MM3	M, 69	IIIa	CRAB	IgGλ
MM4	F, 71	IIb	RAB	IgGκ
MM5	F, 60	Ia	CRAB	IgGκ
MM6	M, 61	IIIa	AB	IgGκ
MM7	F, 43	IIIa	CAB	IgGλ
MM8	M, 80	IIIb	CRAB	IgAλ
MM9	F, 73	Ia	AB	IgGλ
MM10	F, 73	Ia	B	IgGκ light chain disease
MM11	M, 60	IIa	RAB	IgGκ
MM12	M, 33	IIa	AB	IgGκ
D1	F, 64	NA	Neg	Igκ and Igλ
D2	F, 53	NA	Neg	Igκ and Igλ
D3	M, 51	NA	Neg	Igκ and Igλ

MM: MM patient BM sample; D: Donor BM sample

^1^Sex was indicated as M (male) or F (female)

^2^Durie-Salmon staging system [1]

^3^Calcium >2.56 mmol/l; Renal: creatinine >90 μmol/l (F), >110 μmol/l (M); anemia: Hb <12 g/dl (F), <13 g/dl (M); bone lesions [45]

^4^Immunoglobulin G or A, Lambda (Igλ) or Kappa (Igκ) light chain staining of the plasma cells

Approximately 5 ml BM aspirate and 10 ml PB were collected in EDTA tubes from each participant. Samples from eight patients allowed corresponding BM and PB sample analysis, while four additional patients were used for either PB or BM analysis only. Mononuclear cells were isolated using Histopaque-1077 (Sigma-Aldrich, Inc, USA) density-gradient centrifugation and washed with red cell lysis buffer (0.144M NH_4_Cl and 0.01M NH_4_HCO_3_, pH 7.4) for approximately 10 mins at 4°C. Thereafter, the mononuclear cells were fixed and permabilised in 80% cold ethanol and stored at −20°C prior to flow cytometric analysis (BD Biosciences, New Jersey).

### Flow cytometry analysis

Cell-surface monoclonal anti-human antibodies included anti-CD19 phycoerythrin (PE), anti-CD138 allophycocyanin (APC), anti-CD56 fluorescein isothiocyanate (FITC), anti-CD45 peridinin chlorophyll protein (PerCp), anti-CD34 (PE), anti-CD27 (PE), anti-CD19 (APC), anti-CD13 (PE), anti-CD3 (PE), anti-CD33 (APC), anti-CD14 FITC, anti-Ig Lambda (Igλ) (FITC) and anti-Ig Kappa (Igκ) (PE) from BD Biosciences (New Jersey), as well as anti-CD10 (APC), anti-CD20 (APC), anti-CD117 (APC) and anti-Ki-67 (FITC) from Biocom Biotech (South Africa). Nuclear staining antibody for MAGE C1 (ABCAM, UK) was fluorescently labelled using the Mix-n-Stain CF 488A Antibody labelling kit (Sigma-Aldrich, Inc, USA) [22]. A series of antibody panels were used for this study: BM analysis: (1) CD45/CD138/CD56/CD19; (2) CD45/CD138/Ki-67; (3) CD19/CD138/Igκ/Igλ; (4) CD138/CD45/Igκ/MAGE C1; (5) CD45/CD19/CD10/MAGE C1; (6) CD45/CD27/CD20/MAGE C1; (7) CD45/CD34/CD117/MAGE C1 and (8) CD45/CD19/CD34/MAGE C1; PB analysis: (1) CD45/CD19/CD20/MAGE C1 and (2) CD45/CD34/CD19/MAGE C1.

For the analysis using each antibody panel, 2X10^6^ ethanol fixed mononuclear cells were washed twice in 40 ml ice-cold 1X PBS/1% FCS buffer (1500 rpm, at room temperature for 5 mins). Cells were finally re-suspended in 100 μl of cold 1X PBS/0.5% BSA buffer and 5 to 10 μl of each appropriate cell-surface antibody and 5 μg/ml of labelled MAGE C1 antibody were added and incubated at 4°C for 30 mins in the dark. Labelled cells were washed with 1 ml cold 1X PBS/0.5% BSA buffer at 1500 rpm for 5 mins, re-suspended in 1 ml cold 1X PBS/0.5% BSA buffer and kept until flow cytometric analysis.

Quantitative fluorescence analysis was performed with four parameters on the BD Biosciences FACsCalibur using CellQuest software (BD Biosciences) to collect and analyse data. Side scatter (SS) and CD45 staining characteristics were used to first isolate specific leukocyte population groups, which we had previously confirmed with appropriate markers, such as CD14, CD13, CD33, CD19, CD34, CD3, CD10, CD138 [23–24]. These different cell population groups were then selectively gated and the percentage of positive differential B cells, such as stem/immature B lymphocytes, was determined via individual and dual antibody expression. These positive cells for the various stages of B cell maturation were further selectively gated and MAGE C1 expression was determined using histograms and the channel by channel subtraction method [25] with appropriate negative controls (gated areas <10% were regarded as negative for MAGE C1 expression). Additionally donor BM and PB samples were used as controls to exclude all non-specific binding of antibodies to monocytes and granulocytes to ensure no false positive results. A minimum of 300 and 30,000 events per individual antibody and antibody panel, respectively, were analysed with specific gating settings.

### Determination of clonality with IgH specific PCR and capillary electrophoresis

BM samples of patients with proliferating plasma cells were labelled as described above and two populations isolated: CD45^+^/CD19^+^/CD34^+^/MAGE C1^+^ and CD45^-^/CD138^+^/MAGE C1^+^ using the FACS DIVA version 6.1.3 sorter. DNA was released from the sorted cells with heat shock (99°C/5min) and used in a nested PCR to amplify the CDR3 region of the VDJ segment in B cells using characterized primers [26–27]. The PCR conditions were as follows: 1.5 mM MgCl_2_, 0.8 mM dNTPs, 25 pmol FR3A-FITC primer and JH primer, respectively, 3U Hot start GoTaq (Promega, USA) and 1/10^th^ PCR volume of heat-treated cell lysate. Cycle conditions included 95°C for 5 min followed by 45 cycles of 94°C, 55°C and 72°C for 40 sec, respectively, and a final extension of 72°C for 8 min. A second round of PCR was performed using the amplicon of the first PCR as template to ensure optimal amplification. Hi-dye formamide (Applied Biosystems, Life Technologies) and ILS600 (Promega, USA) were added to undiluted PCR products and separated via capillary electrophoresis on the ABI 3130xl (Applied Biosystems) to determine clonality. Controls included extracted DNA from normal PB unfractionated leukocytes and pro-B to pre-B cells from donor BM (polyclonal controls), EBV L1439A (Epstein Barr virus transformed B-lymphocytes) in-house cell line (oligoclonal control), Ramos (ATCC 1596) cell line (monoclonal control) and a no template PCR control.

### Statistical analysis

Statistical analysis of the MAGE C1^+^ expression observed in the different cell populations in the BM and PB of the MM samples was performed using the unpaired 2-tailed Student t-test and the GraphPad Prism 6.05 software (www.graphpad.com/), with *p*-values <0.05 considered statistically significant. The different cell populations with MAGE C1^+^ expression in the MM BM and PB were compared between one another as well as to the corresponding donor BM and PB samples, respectively.

## Results

### Characterization of the BM of the MM patients

Classically, the MM disease is characterised using flow cytometric analysis of the monoclonal plasma cell population in the BM [21,28–30]. In our research population, all the plasma cells in the donor BM samples showed the expected CD45^-^/CD138^+^/CD19^dim+^/CD56^-^/Ki-67^−^ phenotype, while the MM patients BM demonstrated mixed characteristics, including both κ and λ clonality, abhorrent CD56, CD19 and CD117 expression and a variation in the presence of proliferating plasma cells (>20% Ki-67 positivity) (Table 2) [28–31]. Overall, our MM patient population represented a heterogeneous group of different genders, disease types, stages of the cancer as well as varied cell surface characteristics that reflected the abnormal cell-surface antibody staining that is consistent with a MM diagnosis (Tables 1 and 2).

**Table 2:** Summary of plasma cell characteristics in the MM BM study population.

MM patient/Donor number	CD138	CD138/CD19	CD138/CD56	CD138/CD117	CD138/Ki-67
MM1	56*	3	99	90	Neg^†^
MM2	58	3	79	8	Neg
MM3	75	9	99	19	Neg
MM4	50	2	3	4	20
MM5	74	5	74	23	30
MM6	70	5	64	18	59
MM7	77	2	57	72	74
MM8	85	35	79	95	75
MM9	3	2	26	13	89
MM10	14	10	96	32	97
D1	5	6	4	5	Neg
D2	10	16	2	3	Neg
D3	19	11	7	12	Neg

* = Percentage of positive expression of single/dual plasma cell-surface and intracellular antibodies that were used to characterize the plasma cells in the MM and donor samples.

^†^ = <20% Ki-67 positivity.

MM: MM patient BM sample; D: Donor BM sample

### Determination and characterization of the cell phenotype expressing MAGE C1

**Fig 1:**
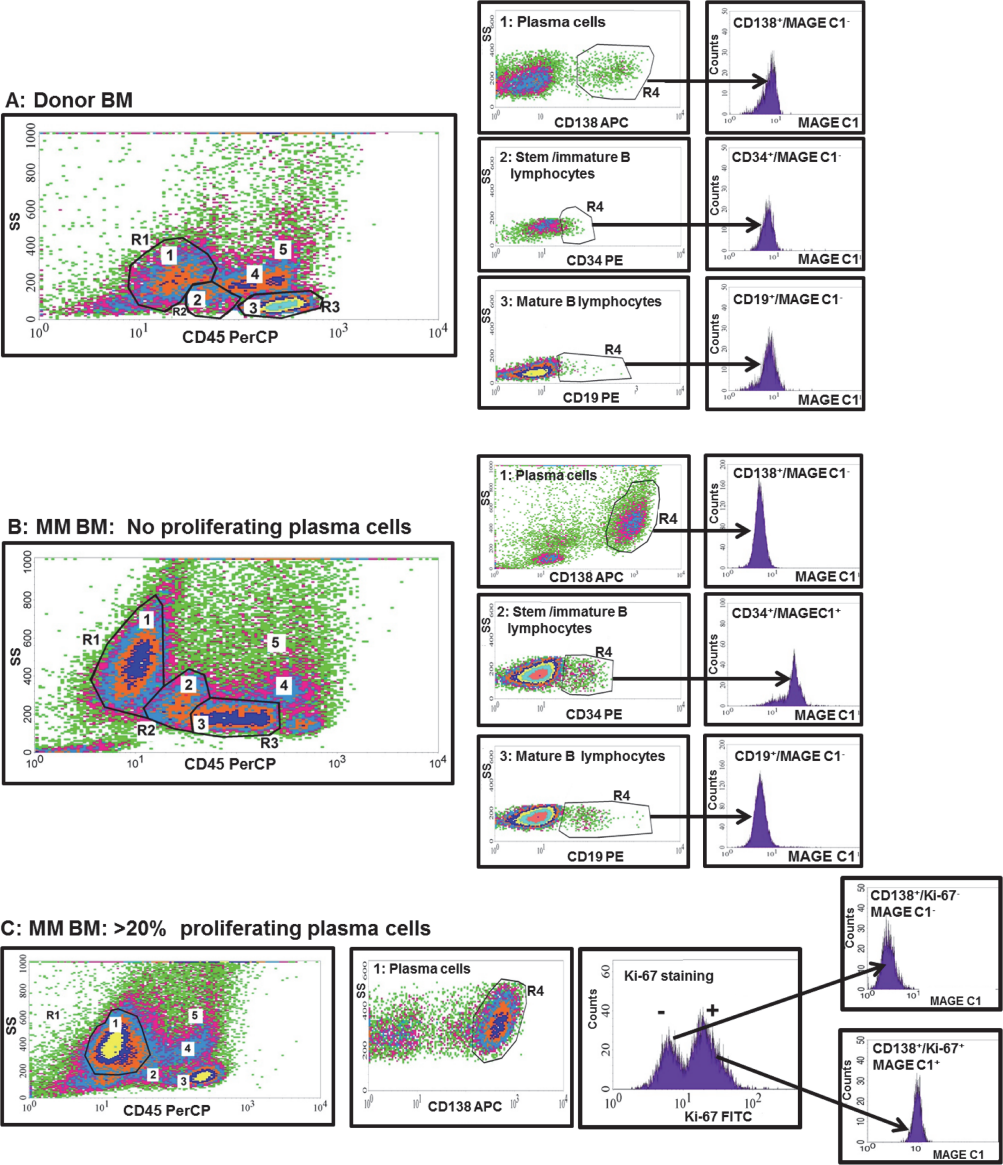
Examples of flow cytometric analysis of the BM of a donor (A) and two MM patients (B and C) to determine the different B cell lineage cell populations expressing MAGE C1. Mononuclear cells from donor and MM patient BM were stained with different antibodies to determine the cell phenotype and stage of B cell maturation where MAGE C1 was expressed. **A and B**: Density plots (SS and CD45) were used to selectively gate around specific cell populations including plasma cells (R1), stem/immature B lymphocytes (R2) and mature B lymphocytes (R3). Using various antibodies specific to the cells of interest gated in R1, R2 and R3, cells with positive antibody expression were further selectively gated (R4) and MAGE C1 expression was determined with histograms. **A**: Donor BM indicated that all selected cell populations were negative for MAGE C1 expression. **B**: MAGE C1 expression was observed in the stem/immature B lymphocytes and not in the non-proliferating plasma or mature B lymphocytes of the MM patient. **C**: Density plots (SS and CD45) were used to selectively gate the plasma cells (R1) and these cells were further confirmed with CD138 (R2). The proliferation index of the plasma cells was determined with Ki-67 expression and this was correlated to MAGE C1 expression via histograms, respectively. MAGE C1 expression was only observed in the proliferating plasma cells (Ki-67>20%). 1: Plasma cells, 2: Stem/immature B lymphocytes, 3: Lymphocytes, 4: Residual monocytes, 5: Residual Granulocytes.

To determine which cellular populations were expressing MAGE C1 in both the BM and PB, a series of antibody panels was used. The initial control donor investigations showed a high degree of non-specific binding of the secondary antibody for MAGE C1 to the monocytic and residual granulocytic populations, despite various blocking strategies. Using antibodies including CD14, CD13, and CD33 the monocytic and granulocytic populations were carefully determined and specific gates were established to exclude all cells contributing to false positivity [23]. Thus using specific gates as well as a combination of SS and CD45 expression to initially identify the cell subgroups of interest, our analysis focused on the plasma cell, stem cell/immature B lymphocyte populations, as well as the mature B lymphocytes (pre-germinal centre) [24]. Based on CD45 expression, bright MAGE C1 positive expression was associated with the stem/immature B lymphocyte cell region in both the MM BM and PB samples of all patients, while a second positive MAGE C1 subpopulation of proliferating plasma cells (dual Ki-67^+^/CD138^+^ phenotype) was observed in six patients in both the BM and corresponding PB sample, as well as three additional patients with unmatched PB/BM samples. This population was identified using a combination of antibody panels 2 and 4 to first selectively gate the proliferating plasma cells in the CD138^+^ population (distinct subpopulation) and then investigate their MAGE C1 expression. Fig. 1 shows examples of the two distinct populations observed in the BM. No significant MAGE C1 expression was observed in any of the donor or PB samples using this protocol, confirming a link to the MM diseased state.

**Fig 2:**
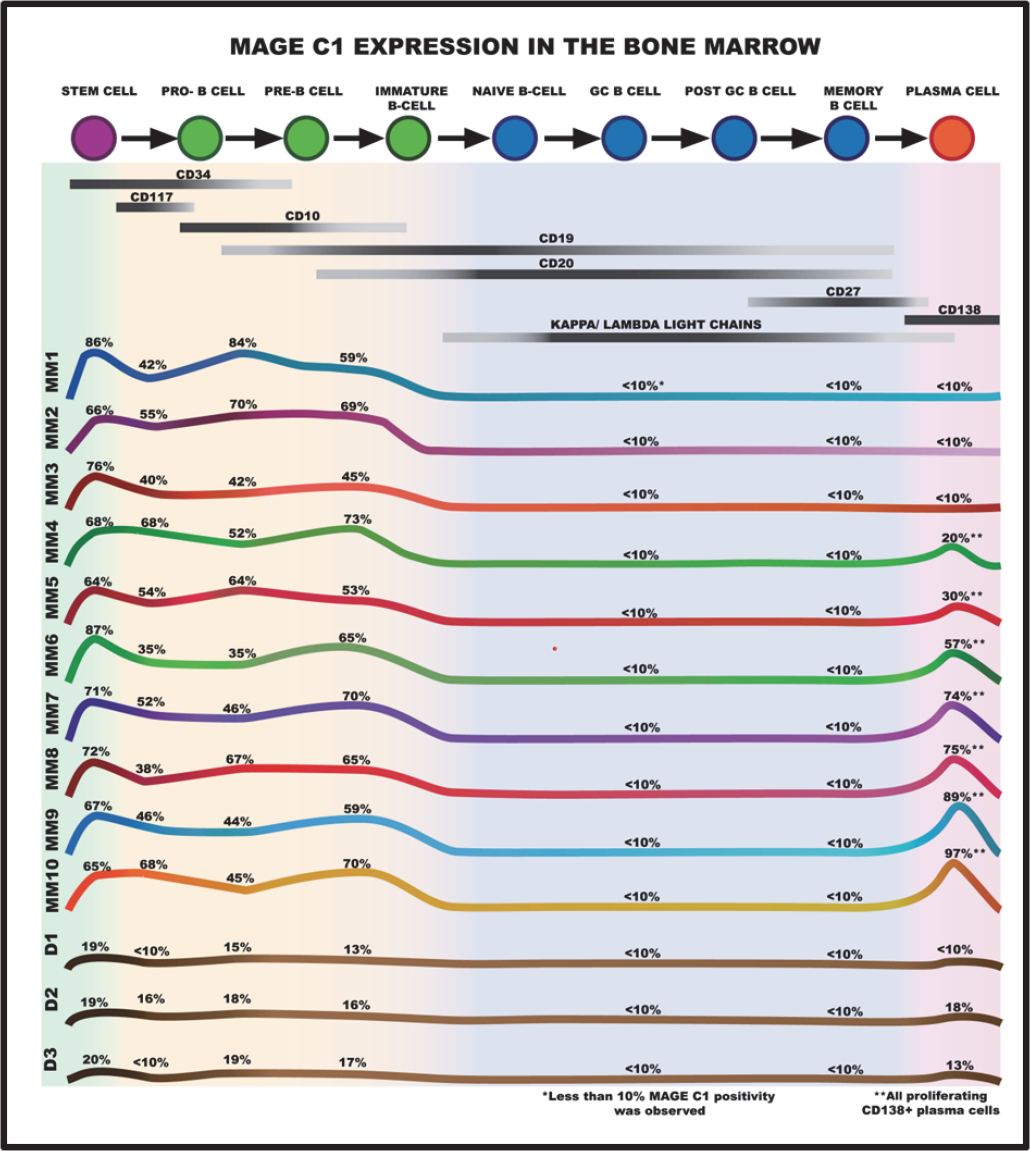
Schematic representation of the co-expression of B cell maturation markers and MAGE C1 in the relevant BM B-lymphocyte sub-populations of MM patients and donors. MAGE C1 expression was observed in the very early stages of B cell maturation involving the CD34^+^, CD117^+^, CD10^+^ and CD19^+^ cells (stem cells to early-mid immature B cells) in the BM, but was absent when light chain expression was expressed (naive and germinal centre B cells). Positive MAGE C1 expression was also observed in the proliferating plasma cell population, but not in the preceding CD27^+^ memory cells present in the BM. The percentage indicated is the number of cells in a specific cell population expressing MAGE C1 (as defined by a specific cell-surface antigen). MM BM samples are ordered according to increasing MAGE C1^+^ expression on the plasma cells. MM 1 to 10 as well as D1 to 3 indicates the different MM patient and donor profiles, respectively.

**Fig 3:**
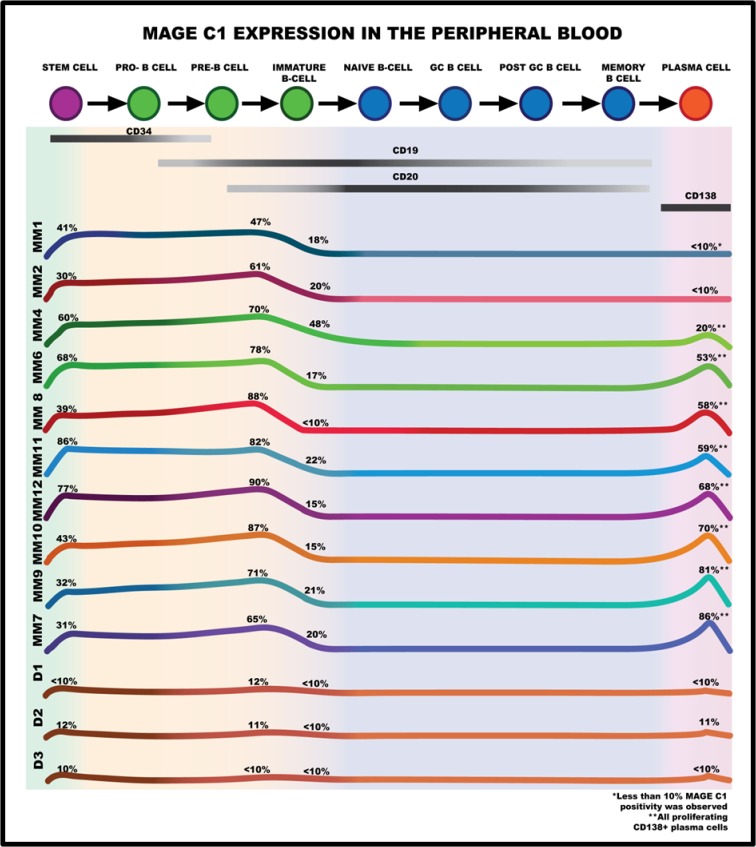
Schematic representation of the co-expression of B cell maturation markers and MAGE C1 in the relevant PB B-lymphocyte sub-populations of MM patients and donors. MAGE C1 expression was observed in the very early stages of B cell maturation involving the CD34^+^ and CD19^+^ as well as the proliferating plasma cells in the PB. Additionally no MAGE C1 expression was observed in the late B cells (CD20^+^) in the PB. The percentage indicated is the number of cells in a specific cell population expressing MAGE C1 (as defined by a specific cell-surface antigen). Kappa/Lambda light chain expression as well as CD117, CD10 and CD27 antibodies were not investigated in the PB. MM 1 to 12 as well as D1 to 3 indicates the different MM patient and donor profiles, respectively.

**Fig 4:**
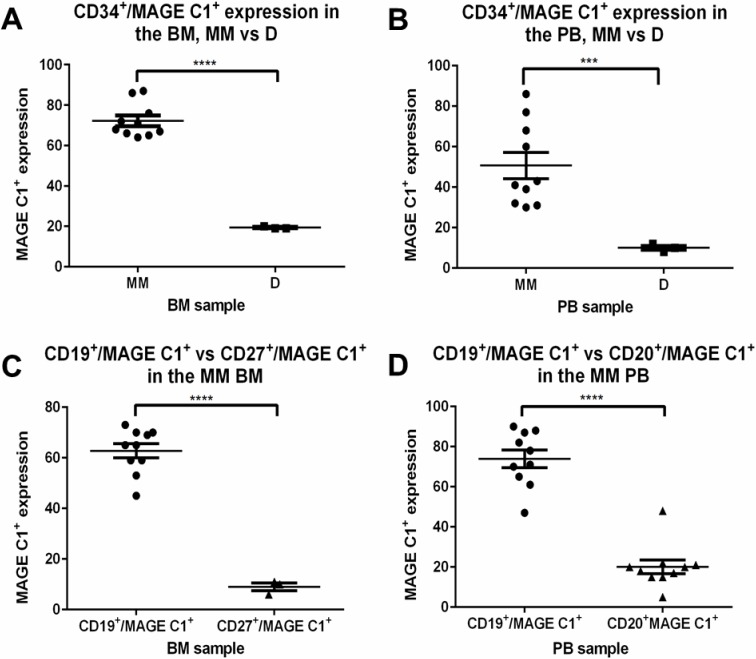
Statistical analysis of MAGE C1 expression in various B cell sub populations. An unpaired 2-tailed Student t-test was used to determine statistical significance between the different cell populations expressing MAGE C1 as indicated, with *p*-values <0.05 considered to be statistically significant. **A**: Comparison of the CD34^+^/MAGE C1^+^ cell population size found in the BM between the MM and donor groups. **B**: Comparison of the CD34^+^/MAGE C1^+^ cell population size found in the PB between the MM and donor groups. **C**: Comparison of the CD19^+^/MAGE C1^+^ and CD27^+^/MAGE C1^+^ cell population sizes in the BM of the MM group. **D**: Comparison of the CD19^+^/MAGE C1^+^ and CD20^+^/MAGE C1^+^ cell population sizes in the PB of the MM group. *** = 0.0001 <*p*< 0.001. **** = *p*-value <0.0001.

To further investigate this CTA expression in the stem/immature lymphocyte B cell sub-populations, MAGE C1 expression was determined along with known markers for the different stages of B cell maturation. These included: CD34, CD10 and CD117 expression for early stem cell/pro-B cells; CD34, CD10 and dim CD19 expression for pro-B to pre-B cells; bright CD19 expression for early to mid-immature B cells; CD20 expression for the late immature to naïve B cells; CD27, dim CD138 and CD20 expression to identify memory B cells and CD138 expression for plasma cells [31,32–34]. Using the various antibody panels and negative controls determined in the validation of the study, the percentage of cells expressing MAGE C1 in both the BM and PB of each MM patient was determined and is presented schematically in Figs. 2 and 3 respectively. While the cell population sizes varied between patients, the relative number of these cells expressing MAGE C1 was fairly constant, and revealed a definite pattern with the expression of this CTA closely linked to B cell maturation in all twelve patients. Importantly, there was a very significant statistical difference in the expression of MAGE C1 between patients with MM and the expression in healthy donor controls. Fig. 4 shows the comparison in MAGE C1 expression between the early stem cell population in both groups (*p*-value<0.001).

As shown in Fig. 2, a very early population of CD34^+^/MAGE C1^+^ cells (CD117^-^/CD19^-^/CD10^-^) was observed in BM samples of all the MM patients, indicating that MAGE C1 was expressed at the very early stages of the B cell lineage. MAGE C1 expression continued in the pro-B to pre-B cells (CD34^+^/CD117^+^ and CD19^+^/CD10^+^) and to the early to mid-immature B cells (CD34^-^/CD19^+^/CD20^dim+^) (Fig. 2). However, analysis of MAGE C1 expression in the late immature and memory cells (CD20, CD27, and Igκ and/or Igλ light chain expression) showed that the protein was no longer being expressed in these cell types, indicating a specific developmental link and a defined malignant cell population. This is clearly demonstrated in Fig. 4, with the highly significant difference in the number of cells expressing MAGE C1 in the CD19^+^ vs CD27^+^ cell populations (*p*-value<0.0001) (Fig. 4).

As mentioned, a second MAGE C1 expressing population was observed in the seven patients that showed the presence of >20% proliferating plasma cells in the BM (Fig. 2). Importantly, this CTA expression was found to be only associated with the proliferating sub-population of plasma cells. A second putative link of MAGE C1 expression to increased proliferation was also observed with a notable increase in the number of proliferating early pro-B to pre-B cells in MM patients BM (sub population demonstrating MAGE C1 positivity) compared to the controls (ave 37% vs 7% (regarded as negative for proliferation)).

Although the cell types present in the PB were more limited, the pattern of MAGE C1 expression was similar to the BM, with circulating stem cells showing positivity (CD34^+^/CD19^-^), as well as early to mid-immature B cells (CD34^-^/CD19^+^/CD20^dim+^) (Fig. 3). As the cells matured further and CD20 expression became more apparent, MAGE C1 was greatly reduced, again reflecting the pattern observed in the BM. Fig. 4 shows the statistically highly significant difference in the numbers of MAGE C1 positive cells in the CD19^+^ population vs the more mature CD20^+^ cells (*p*-value<0.0001). While circulating plasma cells were observed in the PB of all the MM patients, MAGE C1 expression was only apparent in the proliferating plasma cell population if present and corresponded with the expression in the matched BM samples (if applicable) (Fig. 3).

### Determination of clonality of MAGE C1^+^ populations

**Fig 5:**
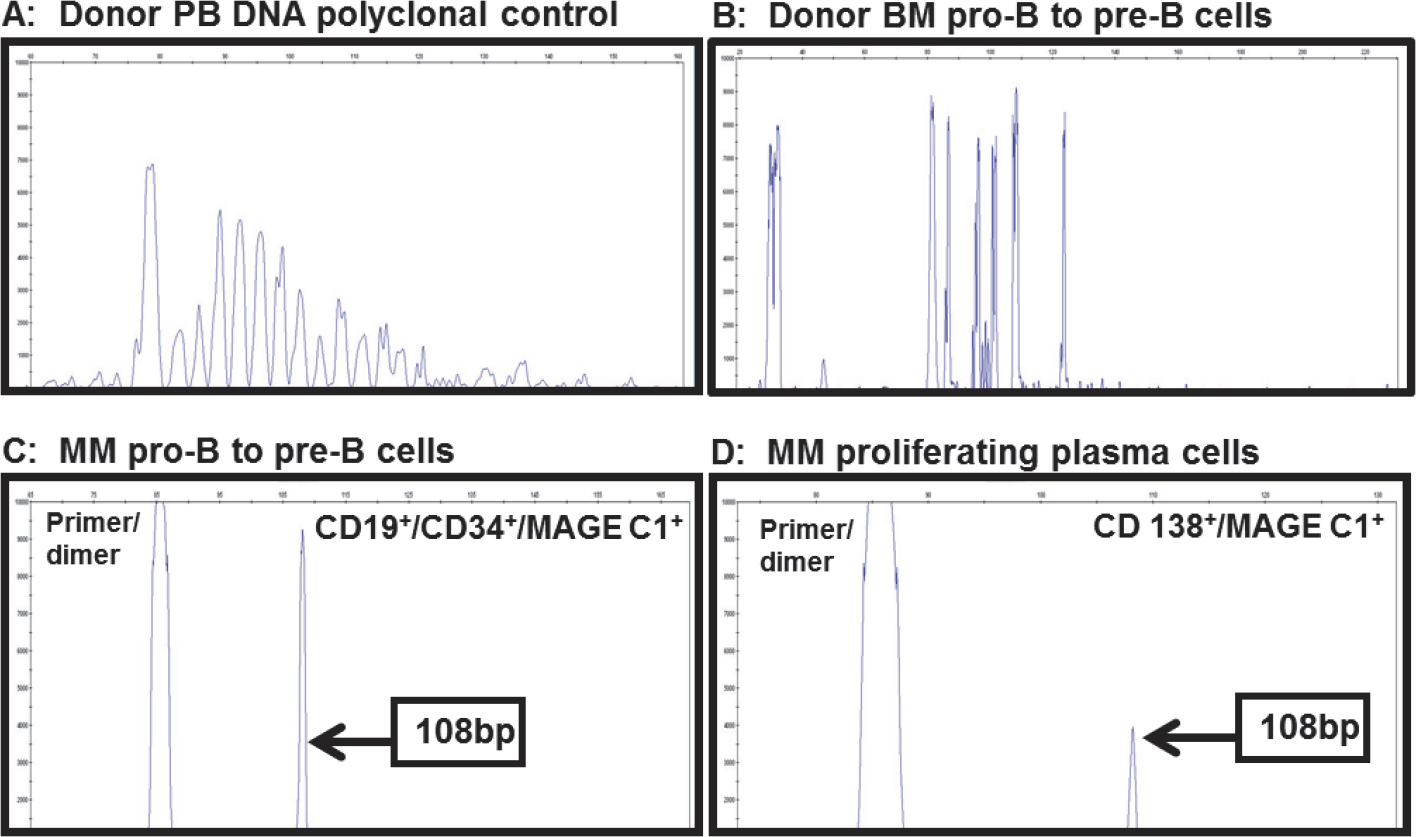
Size clonality analysis of the VDJ region from matched CD19^+^/CD34^+^/MAGE C1^+^ (pro-B to pre-B cells) and CD138^+^/MAGE C1^+^ (proliferating plasma cells) cell populations. Using capillary electrophoresis and specific primers for the CDR3 region cell clonality in MM subpopulations was investigated. As controls, the PB of a donor was analysed (A), as well as the CD19^+^/CD34^+^ isolated population from a donor BM sample (B), both showing polyclonal populations as expected. Comparison of PCR amplicons from the pro-B to pre-B cells and proliferating plasma cells isolated from BM of a MM patient with a large proliferating plasma cell population (MM9, 89%) indicated clonality of these two cell types (C and D).

To investigate the clonality of the proliferating plasma cells expressing MAGE C1 and the MAGE C1^+^ pro-B to pre-B cells, the IgH VDJ region of these cells was analysed for size clonality. Specific primers for the CDR3 region were used on these sorted cell populations to amplify the area of interest, followed by standard capillary electrophoresis analysis. Clonality controls including donor unfractionated leukocytes from PB, pro-B to pre-B cells from donor BM (MAGE C1^−^), an EBV-transformed B lymphocyte cell line and the clonal Ramos cell line, demonstrated the ability of the assay to differentiate between polyclonal, oligoclonal and monoclonal rearrangements in this region, respectively (examples shown in Fig. 5). PCR analysis of the DNA from the CD19^+^/CD34^+^/MAGE C1^+^ (pro-B to pre-B cells) and CD138^+^/MAGE C1^+^ (proliferating plasma cells) sorted cells showed the presence of a single PCR fragment of 108 bp (Fig. 5), which indicated clonality in the two population groups and a strong indicator that they originated from the same malignant clone.

## Discussion

The precursor cell type responsible for disease initiation and ultimately the malignant phenotype in MM remains the most contentious issue. Unfortunately, the lack of a complete understanding of the disease pathogenesis has hampered efforts to effectively treat and cure this fatal disease. In an effort to try to establish which cells carry a malignant signature, we chose to study the expression of MAGE C1 in the B cell lineage of MM patients. MAGE C1 is a CTA whose expression is associated with many malignant phenotypes [15–16] and thus is already established as an abhorrent malignancy marker. It has also been shown to be expressed in most MM cases and linked to the early development of the disease [17–18]. Using a flow cytometric approach we were able to link expression of this malignant marker to two distinct B cell maturation stages, including an early-stem cell-immature B lymphocyte continuum, as well as a defined proliferating plasma cell population in the PB and BM. Importantly, MAGE C1 expression was not observed in any of the corresponding cell populations in samples from healthy donors. Clonality studies indicated a putative common origin of these distinct cell types. Despite the MM patients being heterogeneous in the number of plasma cells, cell-surface characteristics and the number of proliferating plasma cells, the pattern of MAGE C1 expression was similar indicating a common disease mechanism. This novel use of CTA expression in MM has provided a vital link between the various cells of the B cell lineage that are potentially actively involved in MM pathogenesis.

Identification and characterization of MAGE C1 expression specifically to the early stages of the B cell lineage supports the theory that the initial malignant cell phenotype is a primary B cell [7,10–11,35]. Furthermore, high expression of this malignant marker was isolated to the pre-B/early immature B cells, indicating that the initial phenotypic profile of the malignant cell is not a post germinal centre memory cell as suggested by Matsui *et al*. [7–8]. Although clonotypic B cells have been favoured to be the precursory cell in MM, many studies have also indicated that plasma cells are not only responsible for the disease characteristics, but are also actively part of the malignant disease [8–9,36]. Our studies have shown that in MM patients possessing proliferating plasma cells, which is already described as a more aggressive disease [4,6], these plasma cells do indeed have truly malignant characteristics and will actively lead to disease progression or relapse. However, the lack of MAGE C1 expression in the non-proliferating plasma cell bulk in other patients indicates that while these plasma cells cause the symptoms of the disease, they are not involved in the persistence of the disease. The monoclonality of the MAGE C1^+^ pro-B to pre-B cell population compared to the oligoclonal donor pattern, as well as the clonal similarity of the proliferating plasma cells in these patients, is similar to findings in previous studies [3,35,37]. However, we have clearly shown a malignant clonal phenotype (MAGE C1 expression) between the two cell populations, which confirms a common cell of origin for these malignant cells.

This common cell of origin has remained elusive in the past years. Similar to studies relating to other cancers such as myeloid leukaemia [38–39], Matsui *et al*. [8] and Conway *et al*. [10] suggested that the normal stem cell in MM differentiates to a rare cancer stem cell that is responsible for a major component of the disease. Likewise, our results indicate that the stem cells observed in the BM had MAGE C1^+^ expression suggesting that the original stem cell has differentiated to a cancer stem cell and can be the feeder and primary resistant malignant cell phenotype of the downstream B lineage cells. Interestingly, the MAGE C1^+^/CD34^+^ cells (up to 25%) were found in circulation in the PB of the MM patients. This is similar to a study by Szczepek *et al*. [5] where up to 30% of the abnormal stem cells were observed it the PB of MM patients. Therefore, the MAGE C1^+^/CD34^+^ cells found in both the BM and PB of MM patients indicates that the primary malignant cell is not confined to the BM and that migration between these two sights allows for the spread of this cancer.

Controlling the extent of MM is one of the most important aspects of treatment to ensure minimal spreading to other parts of the skeleton. Unfortunately, relapse is commonly observed and studies have shown that clonotypic B lymphocytes can survive current MM chemotherapy treatments [8,10,40]. The most effective treatment involves myeloablative therapy followed by autologous PB blood progenitor cell (PBPC) transplantation [41–42]. However, this treatment involves re-introducing the patient’s CD34^+^ cells back into the BM after chemotherapy, which could potentially be problematic in almost ensuring a relapse, due to the lack of selection against the malignant MAGE C1^+^/CD34^+^ that we identified in this study and their re-introduction via transplantation. This is supported by the finding that in a randomized study comparing disease-free and overall survival rates in patients receiving autologous PBPC transplant of either CD34^+^ selected or unselected PBPCs no significant difference was found between the two groups 37 months post-transplant [42]. Optimal treatment would therefore not only include reducing the plasma cell burden and M-protein levels, but specifically targeting the malignant MAGE C1^+^/CD34^+^ cells before allogeneic transplantation to ensure that malignant cells are not re-introduced into the patients BM.

Determining that MAGE C1 is expressed early in the B cell lineage (pre-B), as well as at the end in the proliferating plasma cells, could indicate a definite role in pathogenesis of the disease. Studies have indicated that MAGE C1 can have a promoting effect on the survival of cells, as well as an association with proliferation genes [6,14]. While we can suggest that MAGE C1 expression may be directly linked to the higher proliferation rate of the early B cells that we observed in this study, further studies would need to be performed to confirm this. Interestingly, MM patients with a high proliferation index have a much more aggressive disease, due to the higher accumulation of plasma cells in the bone marrow [4,6,43]. The re-activation of MAGE C1 expression in these plasma cells may indeed be linked to this proliferation.

The identification of the malignant feeder population in MM through the use of MAGE C1 in this study makes it an interesting target to pursue in targeted cell therapy approaches, such as those employed using MAGE A3 in MM patients [44]. Furthermore, we suggest that MAGE C1 can be used as a molecular marker to monitor patients for MRD using real-time quantitative PCR or flow cytometry by regular monitoring of the various cell populations in the PB of MM patients. This would give a more accurate reflection of disease load and allow more targeted therapy on specific cell populations.
